# EPCs enhance angiogenesis in renal regeneration

**DOI:** 10.18632/oncotarget.10377

**Published:** 2016-07-01

**Authors:** Xin Wang, Yaling Yu, Miaozhong Li, Ali Alkhawaji, Chuan Chen, Xiaolin Liu, Junqun Jiang, Jianse Zhang, Zhibin Wang, Ting Li, Weiwen Zhang, Jin Mei

**Affiliations:** ^1^ Institute of Bioscaffold Transplantation and Immunology, Wenzhou Medical University, Wenzhou, China; ^2^ Medical School of Ningbo University, Ningbo, China; ^3^ Department of Hand Surgery, Ningbo No.6 Hospital, Ningbo, China; ^4^ Anatomy Department, Wenzhou Medical University, Wenzhou, China; ^5^ Department of Anatomy, King Saud bin Abdulaziz University for Health Sciences, Riyadh, Saudi Arabia; ^6^ Department of Medical Neuroscience, Dalhousie University, Nova Scotia, Canada; ^7^ Institute of Neuroscience, Wenzhou Medical University, Wenzhou, China

**Keywords:** endothelial progenitor cells, angiogenesis, decellularized scaffolds, homing, Pathology Section

## Abstract

Decellularized renal scaffolds have previously been used for renal regeneration following partial nephrectomy, in which angiogenesis played a key role. In this study, rats underwent partial nephrectomy and repaired with decellularized renal scaffolds. Subsequently, the labeled EPCs were intravenously injected into rats in EPCs group, and the control group received an equal amount of phosphate-buffer saline (PBS). We chose 1, 2 and 4 weeks post operation as time point. Average microvascular density (aMVD) analyses revealed higher angiogenesis in EPCs group compared with the control group. The expression of angiogenic growth factors including vascular endothelial growth factor (VEGF), platelet derived growth factor (PDGF) and hypoxia-inducible factors 1-alpha (HIF-1α), was generally higher in the EPCs group in all weeks (1, 2 and 4), and peaked in week 2. EPCs were observed to home into renal injury site, promoting angiogenesis across the renal parenchyma-scaffold interface to be potentially used as bridges for EPCs to migrate into the implanted scaffolds. Administration of exogenous EPCs promotes angiogenesis and vasculogenesis in decellularized renal scaffolds-mediated renal regeneration, providing adequate microenvironment for kidney recovery post renal injury.

## INTRODUCTION

Renal tumors can become space-occupying lesions, for which radical nephrectomy, with the requirement of a functioning contralateral kidney, is often performed [1]. However, radical nephrectomy may result in overtreatment, and cannot be appropriate for patients with unilateral renal agenesis (URA) or bilateral renal dysfunction. Partial nephrectomy (PN), therefore, has become an alternative option, allowing the elimination of affected tissue only, to leave as much healthy, functioning tissue as possible [2]. Moreover, advances in imaging and surgical technologies have provided modern tools, enabling precise demarcation and isolation of renal tumors at the interface of renal parenchyma. This less-invasive precision, in turn, has remarkably contributed to increased success rate of partial nephrectomy. Despite the growing favorability of partial nephrectomy, compromised renal functions may still be consequently occurring, [3] imposing the need for an induced renal regeneration for promoted recovery.

The emergence of tissue bioengineering accompanying with the regenerative potentiality of stem cells represents a new era of treatment possibilities. Stem cells have previously been applied to repair renal injuries [4]. Decellularized renal scaffolds have also been demonstrated to be capable of inducing renal regeneration [5]. The regeneration process of which, angiogenesis occurring along the line of renal parenchyma-scaffold interface appeared to be a principal factor in promoting renal regeneration and recovery [4, 6]. Therefore, we speculated that further amplification of angiogenesis with the presence of decellularized renal scaffolds might provide additional chances for better recovery. Angiogenesis have previously been induced using decellularized scaffolds and artificial scaffolds incorporating various growth factors [7-10]. The role of endothelial progenitor cells (EPCs) in angiogenesis has been well established, however, to date, there is no published study that combines decellularized scaffold with EPCs to promote angiogenesis.

This study combines decellularized scaffolds with EPCs to amplify angiogenesis and foster renal regeneration. We constructed renal regeneration animal model as reported in our previous work, then systemically injected the animal with exogenous EPCs through the caudal vein. Observations from this study indicated that angiogenesis in decellularized renal scaffolds-mediated renal regeneration can be greatly enhanced with EPCs. Data collected and subsequent analysis and discussion were intended to provide insights to develop new therapeutic strategies for renal regeneration and enhanced recovery post renal tissue injury.

## RESULTS

### Angiogenesis in renal regeneration mediated by decellularized renal scaffolds

**Figure 1:**
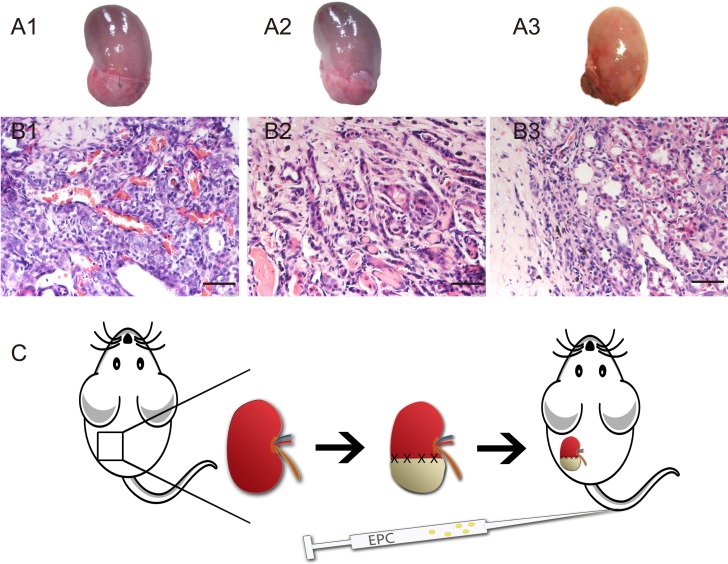
Angiogenesis in decellularized scaffold-grafted kidney **A.** Gross appearances of scaffold grafted-kidney on week 1 (A1), week 2 (A2) and week 4 (A3). **B.** H&E staining of angiogenesis in decellularized scaffold-grafted kidney on week 1 (B1), week 2 (B2) and week 4 (B3). Scale bars = 25 μm. **C.** Design drawing of this research. Left kidney was chose to operate partial nephrectomy. Defect wound was repaired with decellularized renal scaffold. On the operative day, EPCs were injected intravenously.

As our precious study reported, renal regeneration can be induced by decellularized renal scaffold [5]. Macro regrowth was obviously (Figure 1A1-3). Angiogenesis can be observed in renal parenchyma close to the implanted decellularized scaffold. Blood vessels in 4 weeks post operation increased significantly (Figure 1B3 and Figure 6F). To verify our speculation, we created a design drawing (Figure 1C). On the operative day, we injected EPCs intravenously into rats.

### Endothelial progenitor cells (EPCs) culture & co-culture and immunofluorescence characterization

**Figure 2:**
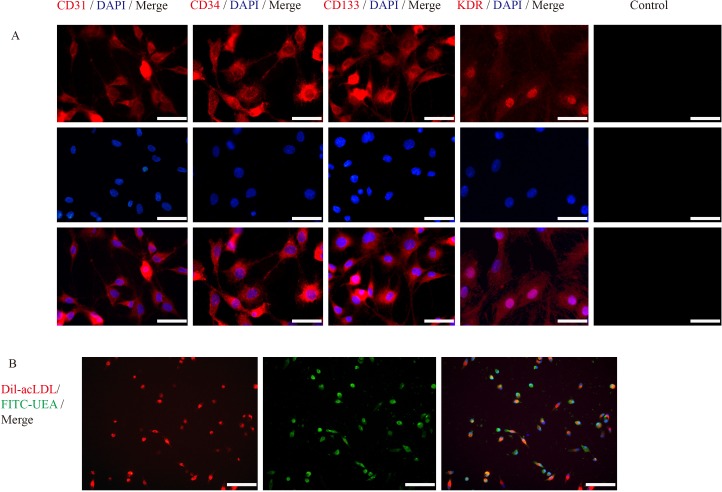
Immunofluorescence characterization of EPCs **A.** Immunofluorescence assay of EPCs revealed the expression of vascular endothelial cells surface biomarkers, CD31, CD34 and CD133 (red) and VEGF (green). Scale bars = 100μm. **B.** Immunofluorescence labeled EPCs: Dil-acLDL (red) and FITC-UEA (green). Scale bars = 50μm.

Mononuclear cells (MNCs) were isolated from femur and tibia bones marrow of SD rats using density gradient centrifugation. These cells were then cultured with EGM-2 MV BulletKit *in vitro*. Around 5 days later, the cells exhibited change in morphology, from round to triangular or polygonal in shape and further changed to spindle-shaped cells 2-3 days later. Ten days post cultivation, immunofluorescence revealed the expression of vascular endothelial cells surface biomarkers; CD31, CD34, CD133 and KDR (Figure 2A). approximately 90% of EPCs used for transplantation, incorporated both DIL-Ac-LDL and FITC-UEA-1 (Figure 2B).

**Figure 3:**
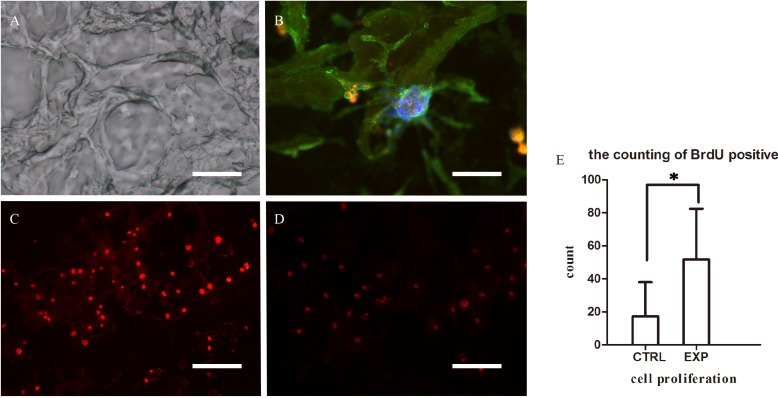
Microscopic characteristics of decellularized scaffolds and EPCs co-cultured with decellularized scaffold **A.** Three-dimensional structure of microvasculature was maintained in decellularized scaffold (100μm). **B.** Immunofluorescence assay of EPCs co-cultured with decellularized scaffold demonstrating adherence to the scaffold. Scale bars: = 25μm **C.**-**E.** Immunofluorescence assay of EPCs demonstrating the expression of BrdU when co-culture with decellularized scaffold (C) and without (D), suggesting that decellularized scaffolds possess the ability to enhance the adhesion and proliferation of co-cultured EPCs. (*P < 0.05) (E). Scale bars: = 50μm.

Bromodeoxyuridine (BrdU) immunofluorescence showed that the number of labeled EPCs greatly increased when co-cultured with slices of decellularized renal scaffold, suggesting that the decellularized renal scaffolds possess an ability to accelerate the cultured EPCs proliferation (Figure 3).

### EPCs trafficking into the scaffold recipient-kidney

Precious studies have shown, stem cells can serve as a therapeutic target for renoprotection and regeneration. Cell therapy has been effective using CD133+ cells derived from bone marrow-derived cells (BMDCs), adipose-derived mesenchymal stem cells, embryonic stem (ES) cells, and induced pluripotent stem (iPS) cells. However, the transplanted EPCs have parallel been detected in lungs and spleens, possibly due to retention or immune surveillance. [11, 12]

**Figure 4:**
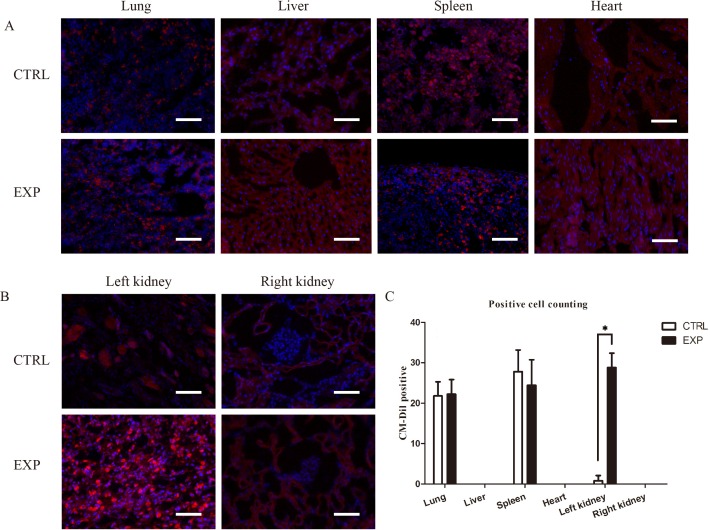
Immunofluorescence tracking of EPCs trafficking **A.** Immunofluorescence assay detection of CM-Dil labeled EPCs (red) in lung, liver, spleen and heart of rats in EPCs and control groups. The labeled EPCs were detected in lungs and spleen of rats in EPCs and control groups without significant differences. **B.** Immunofluorescence assay detection of CM-Dil labeled EPCs (red) in left and right kidneys of rats in EPCs and control groups. A large number of EPCs was found homing to the scaffold recipient-kidney compared to the contralateral intact kidney. However, CM-Dil labeled EPCs were absent in right kidney in both groups (*P<0.05). Scale bar: A-B = 50 μm.

In this study, immunofluorescence reveled CM-Dil positive cells existed during the regeneration process, and EPCs were detected in glomeruli and renal inerestitum of the recipient kidney and in the grafted decellularized scaffold (Figure 4A, 4B). A large number of EPCs was found homing to the scaffold recipient-kidney compared to the contralateral intact kidney. Insignificant numbers of EPCs were detected in lungs and spleen of rats in EPCs and control groups (Figure 4C).

### Expression of angiogenic factors

**Figure 5:**
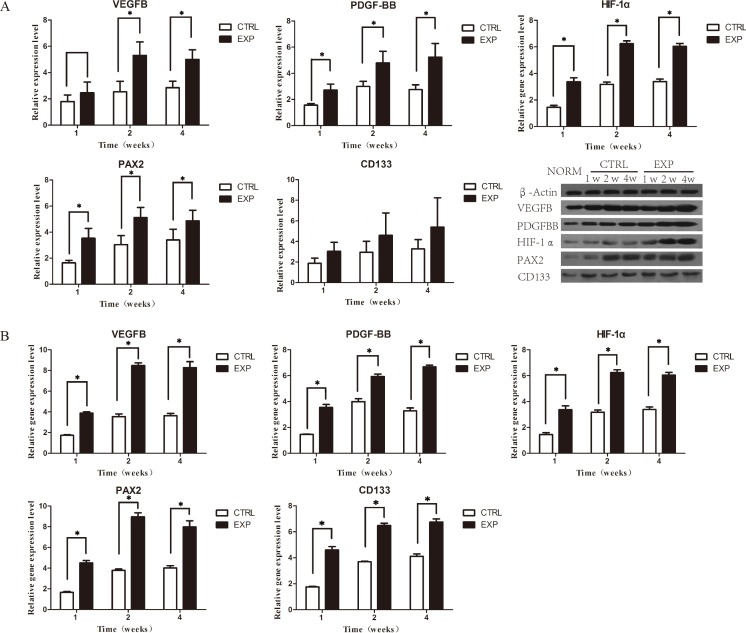
Expressions of related angiogenic growth factors **A.** WB assay showing protein expression of VEGF, PDGF, HIF-1α, Pax2 and CD133 in week 1, 2 and 4 postoperatively. Compared with the control group, PDGF, HIF-1α, Pax2 in EPCs group were significantly expressed in all time points, except VEGF in week 1 and CD133 in week 1, 2, 4. **B.** qPCR of VEGF, PDGF, HIF-1α and Pax2 assay showing significant gene expression of factors in all time points.

Rats from both groups, the EPCs and control, were scarified postoperatively in week 1, 2 and 4 week(s), respectively, and specimens were subsequently obtained from the harvested scaffold recipient-kidneys. VEGF is a pivotal factor in angiogenesis that operates in collaboration with other factors. Western blotting assay (WB) confirmed the expression of vascular endothelial growth factor (VEGF), platelet derived growth factor (PDGF) and hypoxia-inducible factor 1-alpha (HIF-1α). Compared with the control group, WB demonstrated an overall increased protein expression of all factors post to EPCs transplantation; with peak synthesis presenting in week 2. On week 1, PDGF and HIF-1α exhibited significant increased protein expression levels, in particular, VEGF, whereas in week 2 and 4, all factor were significantly expressed (Figure 5A). Quantitative real-time polymerase chain reaction (qPCR) results revealed significant higher gene expression levels of all factors (VEGF, PDGF and HIF-1α) in EPCs group in week 1, 2, and 4 compared to the control group. The gene expression levels of the factors increased remarkably in week 2 and 4 (Figure 5B).

Furthermore, the progression of the renal regeneration was examined. The number of renal progenitor and stem cells were analyzed by paired-box 2 transcription factor (Pax2) and cluster of differentiation 133 (CD133). Pax2 have emerged as a crucial role at multiple steps of kidney development and involves in the action of tubular cell regeneration [13, 14]. Compared to the control group in week 1, 2 and 4, WB assay demonstrated significant increased expression of levels of Pax2 and insignificant increases between protein expressions of CD133 (Figure 5A). Real-time qPCR results revealed significant higher gene expression levels of Pax2 (Figure 5B).

### Average microvessel density (aMVD)

**Figure 6:**
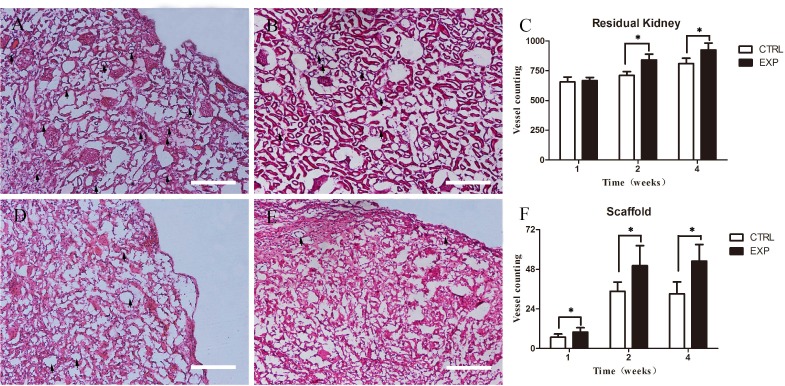
Average microvessel density in residual renal parenchyma and scaffold Residual renal parenchyma in experiment group **A.** show more vessels than in control group **B.** in week 2. Difference results showed in **C.** (*P* < 0.05). And the scaffolds in experiment group **D.** show higher aMVD than the control group **E.** Difference results showed in **F.** (*P* < 0.05). Scale bars = 50μm.

The average microvessel density (aMVD) was evaluated through microscopic examination of hematoxylin and eosin (H&E) staining. Microvessels in residual kidney of the EPCs group exhibited significant higher microvessel formation compared with the control group in week 2 and 4 (Figure 6C). The density of microvessels in residual kidney of both groups in week 1 remained comparable (Figure 6C). Scaffolds of the EPCs group showed significant higher microvessel formation than the control group in week 1, 2 and 4 (Figure 6F).

## DISCUSSION

Regeneration of mammalian kidneys was considered challenging, and compromised renal function secondary to partial nephrectomy was thought to be permanent [15]. We previously reported, decellularized renal scaffolds can mediate renal regeneration for repairing of partially resected kidneys [5]. Data from radionuclide renogram demonstrated enhanced restoration of renal functions. Based on these observations and relying on previous insights [16, 17], we speculated that the intensity of angiogenesis may promote renal regeneration. Impaired organs may be repaired by seeded endothelial cells to help repairing vessels, hence, organs [18]. Furthermore, EPCs have been recognized as an important element in angiogenesis [19, 20], homing into injury sites to participate in vascular repair and angiogenesis [21].

Tissue specimens was H&E stained to evaluate MVD. MVD results revealed higher levels of angiogenesis in EPCs group. These results confirmed that EPCs transplantation can promote angiogenesis in damaged kidney and the grafted scaffolds. WB assay demonstrated an overall increased expression of all factors after EPCs transplantation, with peak in week 2. These findings indicated that EPCs were highly stimulated to transcript these factors in week 2. Observations from qPCR indicated higher gene expression of angiogenic growth factors including VEGF, PDGF and HIF-1α. These observations confirmed that EPCs secret angiogenic growth factors to promote angiogenesis [22].

EPCs used in this study, were derived from MNCs, cultured *in vitro* and labeled with CM-Dil. In confirmation of previous observations [21], immunofluorescence results showed that CM-Dil labeled EPCs were homing to renal injury site. Herein, we speculated that EPCs seemed to promote angiogenesis in the damaged kidney and the renal parenchyma-scaffold interface to be used as bridges for EPCs to migrate to the decellularized scaffolds. Although, small amounts of the labeled EPCs were detected in lungs and spleen, possibly due to retention or immune surveillance [11, 12].

In addition to the present's study, we previously elaborated on the decellularized scaffolds have the capability to support cells adherence and growth. And growth factors, such as PDGF, VEGF and HIF-1α, remained abundantly in decellularized scaffolds. This has chemotaxis effect on EPCs, participating angiogenesis.

## CONCLUSIONS

In this study, we presented a novel therapeutic approach to improve angiogenesis in renal regeneration by using exogenous EPCs. Growth factors, such as PDGF, VEGF and HIF-1α, remained abundantly in decellularized scaffolds. This has chemotaxis effect on EPCs, participating angiogenesis. The combination of EPCs and decellularized scaffold may provide adequate microenvironment for kidney recovery post injury.

## MATERIALS AND METHODS

All experimental protocols were reviewed by the Animal Care Committee at the Ningbo University, and full ethics approval was obtained. Procedures involving animals were performed according to the institutional ethical guidelines for the care and use of animals. A total of sixty male Sprague-Dawley (SD) rats at the age of about 2 month were used in this study. Thirty rats were used for production of decellularized renal scaffolds and EPCs, and thirty rats were divided equally into control and EPC groups (*n* = 15).

### Preparation of decellularized renal scaffolds

Illustrated in our previous work [5, 16, 17, 23].

### Study design

Thirty rats were divided equally into control and EPC groups (*n* = 15). Rats in control and EPCs groups underwent partial nephrectomy and replaced by (equal sized) decellularized renal scaffolds. Rats in the EPC group additionally systemically received about 1×10^6^ EPCs from their caudal vein immediately post decellularized scaffolds grafting.

### EPCs preparation, characterization and transplantation

EPCs were isolated from rats' bone marrow and defined by lineage-markers of endothelial cells (CD31, CD34, VEGFR), stem cell markers (CD133) and characters of taking in (Dil-acLDL and FITC-UEA) [19, 24, 25]. First antibodies as follows: anti-CD31 (1:200; Abcam, UK), anti-CD34 (1:200; Abcam, UK), anti-CD133 (1:200; Abcam, UK) and anti-KDR (1:200; Abcam, UK). EPCs were examined under Olympus fluorescent microscope and images were taken using Olympus soft image viewer.

Prior to injection, EPCs were labeled with CM-Dil (Vybrant™ Dil cell-labeling solution, 50μg/ml; Molecular Probes, USA). EPCs were then washed and suspended in PBS at a concentration of 1×10^6^ /ml. Through a caudal vein, rats in the EPCs group received 1ml of EPCs suspension whereas the control group received an equal amount of PBS.

### EPCs co-culture with the decellularized scaffolds *in vitro*

To confirm the capability of the decellularized scaffolds to improve EPCs adhesion and growth, EPCs were co-cultured with a thin slice of decellularized scaffolds comparing with EGM-2. Proliferation was evaluated with BrdU (Sigma, American). EPCs were seeded onto glass coverslips at a density of 1×10^5^/well in 24-well plate and co-cultured with 100um thick slices of decellularized renal scaffolds and incubated on EGM-2 containing 0.4% FBS for three days. BrdU (20*μ*M) was then added to the culture medium for 1 hour. The specimens were stained with anti-BrdU antibody (1:50, B2631, Sigma). EPCs and its proliferations were observed under Olympus fluorescent microscope and images were captured using Olympus soft image viewer. The intensity of immunoreactivity was quantified using the Image-Pro Plus 6.0 software (Media Cybernetics, USA).

### Specimens harvest

To evaluate the capability of EPCs to improve angiogenesis in the renal regeneration model, angiogenesis and related angiogenic growth factors were evaluated. Rats in both groups were sacrificed postoperatively on week 1, 2 and 4, respectively. The recipient kidneys were harvested and pictures were taken using a digital camera (D3100, Nikon), and specimens were subsequently obtained from harvested recipient kidneys.

### Western blotting assay (kidney)

Total protein was extracted from the scaffold-recipeint kidneys by RIPA lysis buffer (Novland, China), and separated by 10% SDS-PAGE. The specimens were transferred to nitrocellulose membranes (70 V for 1.5 hours). The blots were then blocked in 5% non-fat milk for 1 hour, and incubated with VEGFB, PDGF-BB, HIF-1α, Pax-2 and CD133 primary antibodies overnight at 4°C. Membranes were then washed and incubated with biotinylated secondary antibodies for 2 hrs. Then, the blots were washed again and incubated with NBT/BCIP for 10-30 min. The blots were then scanned and analyzed by GelPro Analyzer software (Media Cybernetics, Silver Spring, MD). Relative expression levels were quantified and normalized to control groups.

### Quantitative real-time polymerase chain reaction (qPCR) TaqMan^®^

To evaluate the gene expression of angiogenic factors, total RNA from the harvested scaffold-recipient kidneys was isolated by lysis in TRIzol (Invitrogen) and PCR was performed as following a specific protocol [24]. The gene expression of VEGFB, PDGF, HIF-1α, Pax-2 and CD133. The primer sequences were shown in Table 1.

**Table 1:** Gene sequences

Gene	Sequences
VEGFB	Forward 5′-GGCCTCTGAAACCATGAACT-3′
	Reverse 5′-ATGCTGCAGGAAGCTCATCT-3′
PDGF	Forward 5′-GGCCTGCAAGTGTGAGACAGTAGTG-3′
	Reverse 5′-TTGAGGTGTCTTGGCTCGATGC-3′
HIF-1α	Forward 5′-AGCTTCTGTTATGAGGCTCACCATC-3′
	Reverse 5′-TCTTCAATGTCAAGATCACCAGCAC-3′
Pax2	Forward 5′-AATCCTGGGCAGGTACTACGAGAC-3′
	Reverse 5′-TGTATTCAGCAATCTTGTCCACCAC-3′
Actb	Forward 5′-CGTAAAGACCTCTATGCCAACA-3′
	Reverse 5′-GGAGGAGCAATGATCTTGATCT-3′

### Average microvessel density (aMVD)

Illustrated in our previous work [16].

### Statistical analysis

All quantitative results were expressed as means + the standard deviation. Independent specimens t-test and one-way anova were used to reveal differences in the level of cytokines and intensity of immunofluorescence among different groups. SPSS software (SPSS Inc., Chicago, USA) was used for analyses, and statistical significance was set at *P* < 0.05.
